# Measurement of Pulmonary Artery Wave Reflection Before and After Mitral Valvuloplasty in Canine Patients With Pulmonary Hypertension Caused by Myxomatous Mitral Valve Disease

**DOI:** 10.3389/fvets.2021.773035

**Published:** 2021-12-02

**Authors:** Tomohiko Yoshida, Kazumi Shimada, Lina Hamabe, Tsuyoshi Uchide, Ryou Tanaka, Katsuhiro Matsuura

**Affiliations:** ^1^Veterinary Centers of America (VCA) Japan Shiraishi Animal Hospital, Saitama, Japan; ^2^Department of Veterinary Surgery, Tokyo University of Agriculture and Technology, Fuchu, Japan

**Keywords:** mitral valvuloplasty, pulmonary artery wave reflection, wave separation analysis, pulmonary hypertension, non-invasive methods of measurement

## Abstract

**Background:** Pulmonary arterial wave reflection provides novel information about pulmonary artery hemodynamics in pulmonary hypertension (PH). PH is common in dogs with myxomatous mitral valve disease (MMVD), though research examining the relationship between pulmonary arterial wave reflection and MMVD with PH is lacking.

**Hypothesis/Objective:** This study investigated conventional echocardiographic parameters and pulmonary artery wave reflection parameters before and after mitral valvuloplasty in canine patients with PH due to MMVD. The parameters were backward pressure (Pb), forward pressure (Pf), and the reflection coefficient calculated as the ratio of peak Pb to peak Pf (RC).

**Animals:** The study subjects were 10 client-owned dogs receiving mitral valvuloplasty for MMVD with PH.

**Methods:** Conventional echocardiographic parameters and pulmonary artery wave reflection parameters were measured before and after mitral valvuloplasty. The relationships between pulmonary artery wave reflection parameters and echocardiographic parameters, estimation of pulmonary artery systolic pressure, and right atrium pressure (RAP) gained by catheter in mitral valvuloplasty were also investigated. Post-operative echocardiography and the measurement of pulmonary arterial wave reflection were performed 2 weeks after mitral valvuloplasty.

**Results:** The parameters of normalized left ventricular internal diameter at end-diastole (LVIDDN), E velocity, and the estimation of pulmonary artery systolic pressure were significantly reduced post-operatively compared with baseline measurements (*p* < 0.05). Post-operative Pb decreased significantly compared with pre-operative measurements (8.8 ± 5.9 to 5.0 ± 3.2 mmHg, *p* = 0.037) as did RC (0.37 ± 0.15 to 0.22 ± 0.11, *p* < 0.01). A statistically significant positive correlation existed between wave reflection parameters and RAP, an estimation of pulmonary artery systolic pressure.

**Conclusions:** Results demonstrate that mitral valvuloplasty can be used to treat secondary PH caused by MMVD, resulting in the improvement of post-operative echocardiographic and wave reflection parameters and a decrease in the right afterload. In some patients, some degree of vascular admittance mismatch persisted, despite the improvement of left atrial pressure. This may be indicative of residual pulmonary arterial disease, which may continue to adversely affect interactions between the right ventricle and the vasculature.

## Introduction

Myxomatous mitral valve degeneration (MMVD) is the most common heart disease in dogs, with severe MMVD causing congestive heart failure, resulting in death within a year (1, 2). Medical treatment has been shown to improve the clinical conditions and prolong the lifespan of dogs with congestive heart failure, as demonstrated in several clinical trials and studies in recent years (3). As medical treatment for MMVD with cardiovascular drugs is palliative, however, MMVD is progressive and the prognosis is poor (2). In addition, persistent increasing left atrial pressure can cause pulmonary hypertension (PH) (4, 5). PH generated from left heart failure is classified into either reactive post-capillary-PH [pulmonary vascular resistance (PVR) ≥2.5 wood units (WU), and transpulmonary pressure gradient (TPPG) >12 mmHg] and passive post-capillary-PH (PVR ≥2.5 WU and TPPG ≤ 12 mmHg) (6–8). Reactive post-capillary-PH is caused by a chronic increase in pulmonary artery pressure, which involves physiologically active substances such as Endothelin-1, and the remodeling of pulmonary artery blood vessels (9, 10). Reactive post-capillary-PH is considered to have a poor prognosis; despite improvements in left atrial pressure through surgery and drug therapy, pulmonary artery pressure is not improved (10–13). Thus, it is very important to detect reactive post-capillary-PH. In human medicine, surgical mitral valvuloplasty (MVP) and valve replacement are performed to radically treat PH caused by left heart failure (14–18). In small animal medicine, MVP is often selected in consideration of the need for permanent antithrombotic therapy and biocompatibility (19). Although the treatment results of mitral valve reconstruction have improved, the prognosis after MVP is poor in cases with reactive post-capillary-PH in which remodeling occurs in the pulmonary artery. Therefore, assessing the pathophysiology of pulmonary hypertension is very important. In recent years, several studies have reported that pulmonary arterial wave reflection may provide additional information about right ventricular afterload and can be a useful indicator in assessing the pathophysiology of pulmonary hypertension (20, 21). Pulmonary artery wave reflection occurs when the forward blood flow out the right ventricle is reflected by the pulmonary arterial tree, generating a backward wave (22, 23). The method of estimating the magnitude of pulmonary artery wave reflection is to separate pulmonary artery pulse pressure into its forward and backward components by wave separation analysis (24, 25). This analysis requires measurements of both pressure and flow velocity waveforms simultaneously (20, 25). A previous paper proposed that measuring pulmonary artery wave reflection can be carried out non-invasively by estimating the pulmonary artery pulse pressure from tricuspid regurgitation (TR) velocity and evaluating the pattern of flow profile at the right ventricular outflow tract (RVOT). In addition, the pulmonary artery wave reflection gained by Doppler echocardiography also can be related to the prognosis of PH in a small animal clinical environment (26).

This study evaluated the change in pulmonary artery wave reflection before and after MVP in canine patients with PH due to MMVD. In addition, we investigated the relationship between the pulmonary artery wave reflection and cases with residual post-operative PH by measuring pulmonary artery wave reflection before and after MVP in patients with PH due to MMVD.

## Materials and Methods

### Animals

The study was carried out at a private clinic (SHIRAISHI Animal Hospital, Saitama, Japan) between August 2019 and April 2021. The subjects were 10 client-owned dogs receiving MVP exclusively for MMVD with suspected PH due to left heart disease. Permission for this study was granted by SHIRAISHI Animal Hospital Animal Care and Use Committee (protocol number R2-062). Before subject enrollment, written, informed client consent was obtained. All cases were classified with American College of Veterinary Internal Medicine (ACVIM) stage C or D, as determined through comprehensive evaluation including medical history and records, physical examination, hematology, serum biochemistry, diagnostic electrocardiography, radiography, and echocardiography. MVP with cardiopulmonary bypass was performed in all cases.

The definitive diagnosis of PH requires right heart catheterization (RHC). However, given RHC is difficult to apply in small animals. Therefore, PH were suspected if a maximal TR velocity of ≥3.4 m/s was observed, based on ACVIM consensus, and if the dogs presented with PH-related clinical signs. PH was also suspected if a maximal TR velocity of ≥2.9 m/s was observed via Doppler echocardiographic assessment, or if other echocardiographic findings suggestive of PH were found in dogs with PH-related clinical signs. Other echocardiographic findings were defined as the flattening of the interventricular septum, especially systolic flattening, pulmonary artery enlargement, or systolic notching of the Doppler RV outflow profile (6). Clinical signs considered potentially related to PH included: syncope without another identifiable cause; respiratory distress at rest; activity or exercise terminating in respiratory distress; abdominal distention due to ascites; tachypnea at rest; increased respiratory effort at rest; prolonged post-exercise or post-activity tachypnea; and cyanotic or pale mucous membranes (6). If the patient included above criteria after surgery, we judged the patient to be persistent PH.

The main causes of precapillary (heartworm disease, pulmonary embolism, chronic respiratory diseases, etc.) were excluded using blood examination, ultrasonogram, thorax radiography as much as possible without hemodynamic examination by catheter.

### Study Protocol

Conventional echocardiographic parameters and pulmonary artery wave reflection parameters were measured before and after MVP. The relationships between pulmonary artery wave reflection parameters and echocardiographic parameters, right atrium pressure (RAP) gained by catheter, and pulmonary artery systolic pressure gained by echo-Doppler were also investigated. Echocardiography and measurement of pulmonary artery wave reflection were performed 2 weeks after MVP.

### Principles Underlying the Echo-Doppler Method of Assessing Pulmonary Artery Wave Reflection

Pulmonary artery wave reflection can be obtained by simultaneously measuring pulmonary artery pressure and flow velocity. Previous research, however, reported that wave reflection as measured by Doppler echocardiography correlated with wave reflection measured by catheter, indicating that wave reflection can also be measured in this manner. The pulmonary artery wave reflection was calculated by measuring the TR flow and RVOT using Doppler echocardiography by the same method as in the previous paper (26). The method of calculating pulmonary artery wave reflection from Doppler echocardiography is briefly described; pulmonary artery wave reflection can be analyzed based on the concept of wave intensity analysis, which determines the origin, type, and timing of traveling waves in circulation by combining measurements of pressure (P) and velocity (U) (27). This allows the wave to be separated into forward-traveling and backward-traveling components (24, 27, 28). Wave speed, which represents the local elastic properties of the artery, can be calculated by the P-U loop method (29). This takes advantage of the water hammer equation for the relationship between P and U under conditions with no wave reflection.


(1) $$\begin{array}{l} \text{c }=\;\left(dP\;/\;dU\right)\;/\;\rho \end{array}$$

where dP is the temporal change in P, dU is the temporal change in U, ρ is the blood density (1,050 kg/m^3^), and c is the wave speed. Pressure attributed to forward-traveling (forward pressure; Pf) and backward-traveling (backward pressure; Pb) waves can be separated using Equations (2) and (3). Backward-traveling waves indicate pulmonary artery wave reflection.


(2) $$\begin{array}{l} \text{dPf }=\;\left(\text{dP }+\;\rho \text{c dU}\right)\;/\;2 \end{array}$$

(3) $$\begin{array}{l} \text{dPb }=\;\left(\text{dP }-\;\rho \text{c dU}\right)\;/\;2 \end{array}$$

where dPf is the temporal change in Pf and dPb is the temporal change in Pb. Pf and Pb can then be determined as the total sum of these differences.


(4) $$\begin{array}{l} \text{Pf }=\text{ ΣdPf} \end{array}$$

(5) $$\begin{array}{l} \text{Pb }=\text{ ΣdPb} \end{array}$$

The non-invasive method proposed for the assessment of pulmonary artery wave reflection adopts echo-Doppler-derived P and U values rather than direct measurements. The P waveform is estimated from a continuous-wave Doppler tracing of TR, using the simplified Bernoulli equation. Once the right atrial pressure was assumed to be constant, the temporal change in P (dP) could be calculated using only the instantaneous pressure gradient of TR. In contrast, the U waveform was obtained by measuring flow at the right ventricular RV outflow tract (RVOT), using pulsed-wave Doppler. The calculations were performed throughout the ejection period. Wave separation analysis is performed on the pulse pressure and flow velocity waveforms by synchronizing waveforms of TR flow and RVOT flow using electrocardiogram. This analysis resulted in the determination of three wave reflection indices: Pb, Pf, and the reflection coefficient (RC), which was calculated as peak Pb/peak Pf. These waveforms were smoothened using a Savitzky–Golay filter and then ensemble-averaged over three cardiac cycles with reference to the R wave on the electrocardiogram. The calculations were performed using an in-house program code, written in MATLAB (MathWorks 2019b, Massachusetts, USA).

### Echocardiography

The echocardiographic assessment was carried out while the subject was maintained in lateral recumbency and examined under standard conditions, using an Aplio^TM^ 300 with a sector probe of five MHz (Canon medical system, Tokyo, Japan). Analysis of pulmonary artery wave reflection was also performed by same echocardiography machine. Three consecutive heartbeats were recorded at the end of the expiratory phase. To evaluate structural and functional changes during the course of reverse remodeling of the left atrium and the left ventricle, combined conventional echocardiography protocol including Two-dimensional, M-mode, Doppler blood flow, and tissue Doppler imaging techniques from the right and left parasternal long- and short-axes views were carried out (30, 31). The following parameters were measured at each time interval: normalized left ventricular internal dimension in diastole (LVIDDN); the ratio of the left atrial dimension to the aortic annulus dimension (LA/Ao); fractional shortening (FS); early diastolic mitral inflow (E) velocity; the ratio of peak velocity of early diastolic transmitral flow to peak velocity of late diastolic transmitral flow (E:A); systolic (S′), early diastolic (E′), and late diastolic (A′) wave signals as measured by Tissue Doppler imaging at septum (sep) and left ventricular lateral (lat) wall, respectively. LVIDDN was calculated from the left ventricular internal dimension in diastole (LVIDd) and the bodyweight was measured concurrently by an established allometric formula (30). TR velocity, obtained by continuous-wave Doppler, was measured from the view that allowed the clearest envelope of the TR velocity and maximum speed (6). The flattening of the interventricular septum was identified on M-mode images from the right parasternal short-axis view (32). The RVOT and main pulmonary artery-to-aortic root diameter ratio (MPA:AO) were measured from the standard right parasternal short-axis view (2, 33). RVOT flow was assessed with pulse-wave Doppler and obtained by placing the sample volume (2 mm) centrally between the opened pulmonary valve leaflets. Ejection time (ET), acceleration time (AcT), and AcT:ET ratio were assessed using RVOT flow profiles as follows. The AcT was measured as the time between the onset of the Doppler flow signal to the peak flow velocity. ET was measured from the onset of the Doppler RVOT signal to the end of the signal, and the AcT:ET ratio was calculated (33, 34). In all dogs, the echocardiographic characterization of the RA and RV were obtained from the left apical 4-chamber view optimized for the right heart. The right atrial area (RAA) was measured by planimetry at the end of the ventricular systole tracing from the lateral aspect of the tricuspid annulus to the septal aspect, excluding the area between the leaflets and annulus, following the RA endocardium, and excluding the caudal vena cava, cranial vena cava, and RA appendage. The RAA index was calculated as RAA divided by body surface area (BSA). Right ventricular end-diastolic area index (RVEDA) was measured by planimetry at the end of ventricular diastole, tracing from the lateral aspect of the tricuspid annulus to the septal aspect, excluding the area of the annulus and trabecular structures, following the RV endocardium. The RVEDA index was calculated as the ratio of RVEDA and BSA. The BSA was calculated as following equation.


$$\begin{array}{l} \text{BSA}=\;0.101\;\times \text{ body weight }{\left(\text{kg}\right)}^{2/3} \end{array}$$

The end-diastolic MPA diameter was measured just below the closed pulmonary valve, with the aortic diameter being measured from the same view, and through this the MPA:AO ratio was calculated. Pulmonary artery systolic pressure (sPAP) was estimated by applying the simplified Bernoulli equation to a continuous-wave Doppler tracing of TR flow and adding a term of right atrial pressure as below.


$$\begin{array}{l} \text{Estimated sPAP }=\;4\;\times \;\left(\text{TR velocity}\right)2\;+\text{ RAP} \end{array}$$

The RAP was measured at the time of MVP invasively.

### Surgical Procedure

Atropine (Atropine Sulfate injection; Mitsubishi Tanabe Pharma Corporation, Osaka, Japan, 0.05 mg/kg, SC), fentanyl (Fentanyl Citrate; Daiichi Sankyo Company, Limited, Tokyo, Japan, 5 μg/kg, IV), and midazolam hydrochloride (Dormicum; Astellas Pharma Inc., Tokyo, Japan, 0.2 mg/kg, IV), were administered pre-operatively. Anesthesia was induced with propofol 1% (Propofol Mylan; Mylan Seiyaku, Tokyo, Japan, 6 mg/kg bolus, IV) and maintained with 1–2 vol% of isoflurane (Isoflurane for Animal Use; Intervet, Osaka, Japan) in 100% oxygen.

The patient was placed in right lateral recumbency. The right femoral artery and vein were cannulated for measurement of atrial pressure (RAP). After patients were heparinized at a dose of 400 U/kg, the left carotid artery and jugular vein were cannulated for cardiopulmonary bypass (CPB). activated clotting time was used for determining the anticoagulant effect of heparin. Cardiac arrest was achieved by aortic clamping followed by the administration of cardioplegia from an aortic root cannula. MVP was performed as previously described. In brief, the left atrium was approached through a fifth intercostal thoracotomy and left auricle incision. The MVP is consisted of artificial chordae tendineae reconstruction and mitral annuloplasty, using expanded polytetrafluoroethylene. After the closure of the left atrium, the aorta was unclamped and sinus rhythm was restored. Patients were weaned from CPB after conducting modified ultrafiltration and confirming the stability of the hemodynamic system. A constant-rate infusion of protamine 4 mg/kg was administered for 30 min through the cephalic vein. The activated clotting time was measured after the end of the protamine infusion. After confirming that the protamine was antagonized, the chest was closed according to the standard method, and the crotch and cervical arteries and veins were anastomosed to finish the operation.

### Statistical Analysis

Continuous data are expressed as the mean ± standard deviation (SD). Categorical data are expressed as a number and percentage. The level of significance was set to *p* < 0.05. The normal distribution of the data was evaluated using a Kolmogorov–Smirnov test. The assumption of homogeneity of variances was determined by Bartlett's test. For normally distributed parameters, differences between groups were analyzed using a paired *t*-test. For non-parametric statistics, differences between groups were analyzed using a Wilcoxon matched-pairs signed-rank test. Spearman's correlation coefficients and multivariate linear regression analysis were used to assess the relationship between the pulmonary artery wave reflection parameters and echocardiographic parameters, and the hemodynamic parameters gained by catheter. Statistical analyses were performed using GraphPad Prism 8.0 (GraphPad Software, San Diego, California, USA).

## Results

### Study Population

A total of 10 dogs were the subject of this study, aged 9.6 ± 2.3 years old (range 7−14 years), and with a bodyweight of 4.6 ± 2.1 kg (range 2.4−10 kg). Breeds were Cavalier King Charles spaniel (*n* = 1), Chihuahua (*n* = 4), Maltese (*n* = 1), miniature dachshund (*n* = 1), mixed-breed (*n* = 2), Shiba Inu (*n* = 1). In accordance with the ACVIM classifications scheme, seven and three dogs were diagnosed with stage C and D ACVIM, respectively. When first diagnosed, 30% of patients had syncope, 20% of patients had ascites and/or pleural effusion. In addition, 20% of PH cases presented with septal flattening, and 30% had a notch in the pulmonary artery waveform at the initial visit. Before surgery, pimobendan and a diuretic drug had been prescribed in all dogs and sildenafil had been prescribed in only one dog. All canine subjects were discharged post-operatively within 2 weeks and in medication affecting hemodynamics (diuretics, inotropes, etc.) were discontinued. However, after hospitalization, two cases were prescribed sildenafil 0.5 mg/kg BID (sildenafil: Camber Pharmaceuticals, Inc., New Jersey, USA) due to presenting symptoms and echocardiography findings associated with PH. Two dogs with persistent PH after MVP presented with the flattening of the interventricular septum, however, TR was mild (TR velocity 3.0 m/s in both PH patient).

### Conventional Echocardiographic Parameters Before and After MVP

**Figure 1:**
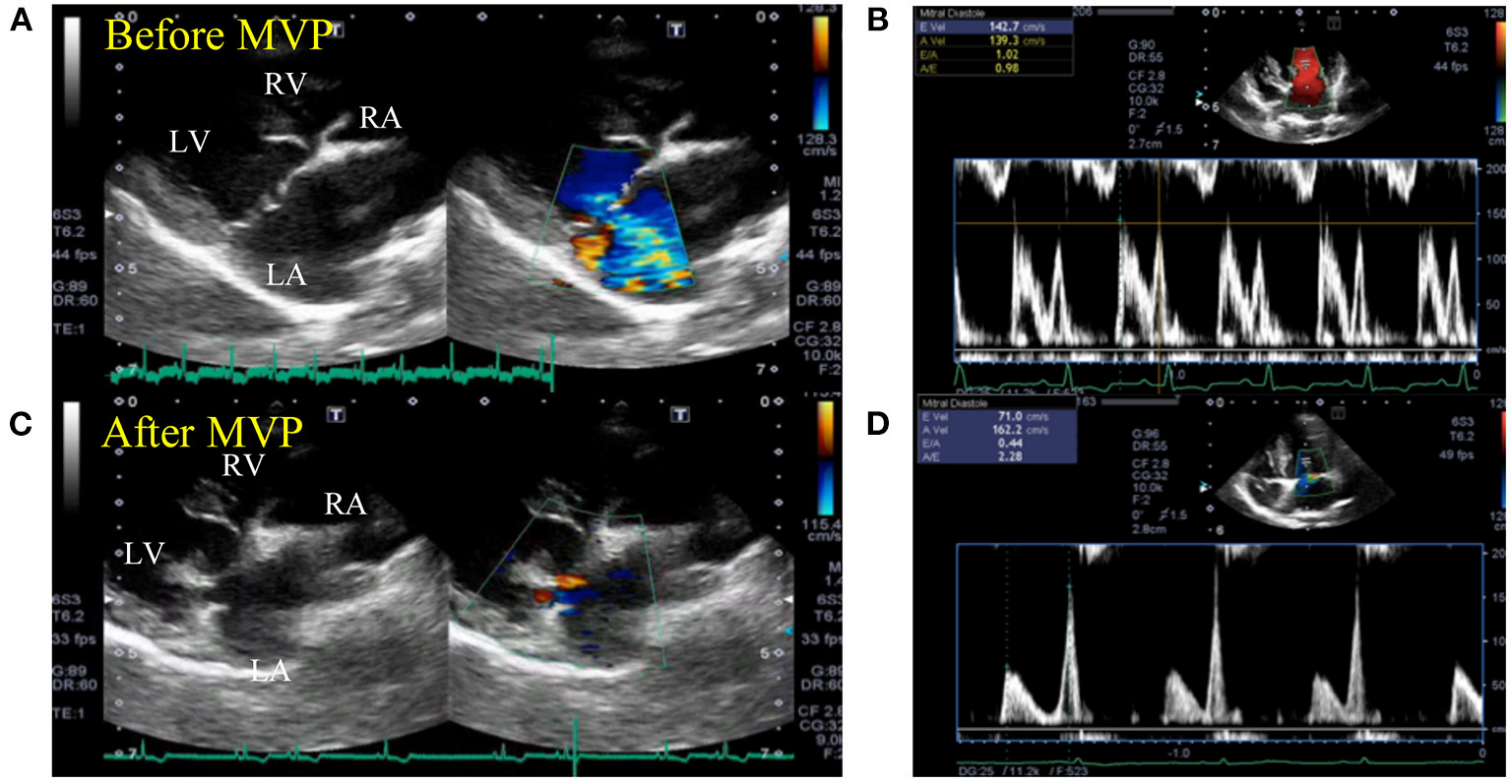
Representative echocardiogram images before and after mitral valvuloplasty. Two-dimensional echocardiography at the long-axis LV inflow view showing the enlarged left ventricle and atrium chamber **(A)** and elevated mitral inflow velocity **(B)** before mitral valvuloplasty. The cardiac functional parameters were greatly restored, with reduced left ventricular and atrium lumen size **(C)** and mitral inflow velocities **(D)** after mitral valvuloplasty. LA, left atrium; LV, left ventricle; RA, right atrium; RV, right ventricle.

Conventional echocardiographic data before and after MVP are summarized in Table 1. Compared with baseline measurements, the following were significantly reduced (*p* < 0.05) post-operatively: LVIDd, LVIDDN, LA/Ao, FS, E velocity, E′ sep, E′ lat, A′ lat, RVOT, and TR velocity. Echocardiography revealed a reduction in the left-sided chamber size and stable mild mitral regurgitation after MVP in all dogs (Figure 1).

**Table 1:** Conventional echocardiography parameters before and after mitral valvuloplasty.

**Conventional echo parameter**	**Pre-operative**	**Post-operative**	***P*** **-value**
LVIDd, mm	34.6 ± 5.1	23.6 ± 4.05	<0.01
LVIDDN	2.27 ± 0.26	1.52 ± 0.19	<0.01
LA/Ao	2.33 ± 0.28	1.52 ± 0.18	<0.01
FS (%)	49.4 ± 6.2	32.6 ± 6.1	<0.01
E velocity, cm/s	139.3 ± 24.2	77.98 ± 13.3	<0.01
E:A	1.5 ± 0.42	0.77 ± 0.22	<0.01
E′ sep, cm/s	8.6 ± 2.6	5.3 ± 1.67	0.04
A′ sep, cm/s	6.9 ± 0.73	5.5 ± 2.28	0.08
E:E′ sep, cm/s	17.2 ± 4.6	16.8 ± 6.23	0.7
E′ lat, cm/s	8.7 ± 1.6	5.47 ± 1.07	<0.01
A′ lat, cm/s	7.9 ± 2.7	4.9 ± 1.55	<0.01
E:E′ lat, cm/s	16.8 ± 6	15.03 ± 3.13	0.56
RVOT velocity, cm/s	78.6 ± 19.1	106 ± 19.1	0.04
AcT:ET	0.4 ± 0.09	0.52 ± 0.05	0.16
MPA:Ao	1.02 ± 0.13	1.01 ± 0.1	0.39
TR velocity, m/sec	3.79 ± 0.49	2.81 ± 0.39	<0.01
RAA index	7.7 ± 3.5	6.6 ± 1.7	0.69
RVEDA index	10.0 ± 3.5	8.9 ± 3.1	0.37

*Data are expressed as means ±. P-values represent significant statistical differences between pre-operative and post-operative echo parameters.*

*A′, late diastolic wave signal as measured by Tissue Doppler imaging; E velocity, early diastolic mitral inflow velocity; E′, early diastolic wave signal as measured by Tissue Doppler imaging; E:A, the ratio of peak velocity of early diastolic transmitral flow to peak velocity of late diastolic transmitral flow; FS, fractional shortening; LA/Ao, the ratio of the left atrial dimension to the aortic annulus dimension; lat, mitral annulus at the left ventricular lateral wall; LVIDd, left ventricular internal dimension in diastole; LVIDDN, normalized left ventricular internal dimension in diastole; MPA/AO, main pulmonary artery/aorta; RAA index, right atrial area index; RVEDA index, right ventricular end-diastolic area index; RVOT, right ventricular outflow tract; S', systolic wave signal as measured by Tissue Doppler imaging; sep, mitral annulus at the septal wall; TR, tricuspid regurgitation*.

### Pulmonary Artery Wave Reflection Parameters Before and After MVP

**Figure 2:**
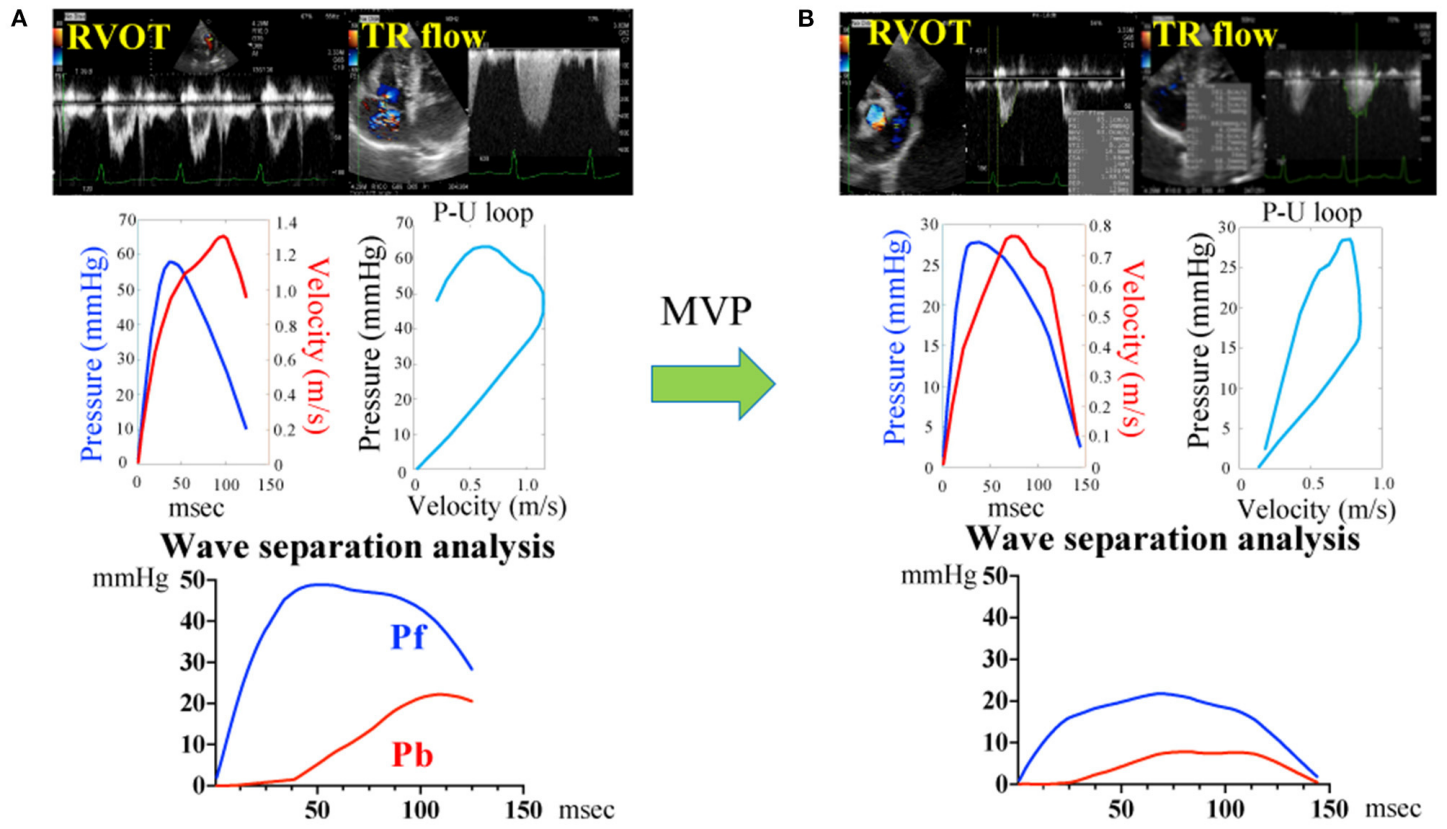
Pulmonary artery wave reflection measured by representative original trace using Doppler echocardiography before **(A)** and after **(B)** mitral valvuloplasty. Pressure (P) information was gained from the TR flow using the Bernoulli equation and the flow velocity (U) information was gained from the RVOT. Determination of the P-V loop from P and U. Pulmonary artery wave reflection parameters were gained by wave separation analysis. Pf, forward pressure; Pb, backward pressure; P-U loop, pressure-flow loop; RVOT, right ventricular outflow tract; TR, tricuspid valve regurgitation flow.

Figure 2 and Table 2 show the representative assessment method of pulmonary artery wave reflection, and pulmonary artery wave reflection parameters before and after MVP. Post-operative Pb and RC decreased significantly compared with pre-operative measurements (Table 2, Pb decreased from 8.8 ± 5.9 to 5.0 ± 3.2 mmHg, *p* = 0.037; RC also decreased from 0.37 ± 0.15 to 0.22 ± 0.11, *p* < 0.01).

**Table 2:** Wave reflection parameters before and after mitral valvuloplasty.

**Wave reflection parameter**	**Pre-operative**	**Post-operative**	***P*** **-value**
Forward pressure (Pf), mmHg	23.6 ± 11.7	21.0 ± 6.4	0.77
Backward pressure (Pb), mmHg	8.8 ± 5.9	5.0 ± 3.2	0.037
Reflection coefficient (RC = Pb:Pf)	0.37 ± 0.15	0.22 ± 0.11	<0.01

*Data are expressed as means ±. P-values represent significant statistical differences between pre-operative and post-operative wave reflection parameters. Pb, backward pressure; RC, reflection coefficient*.

**Figure 3:**
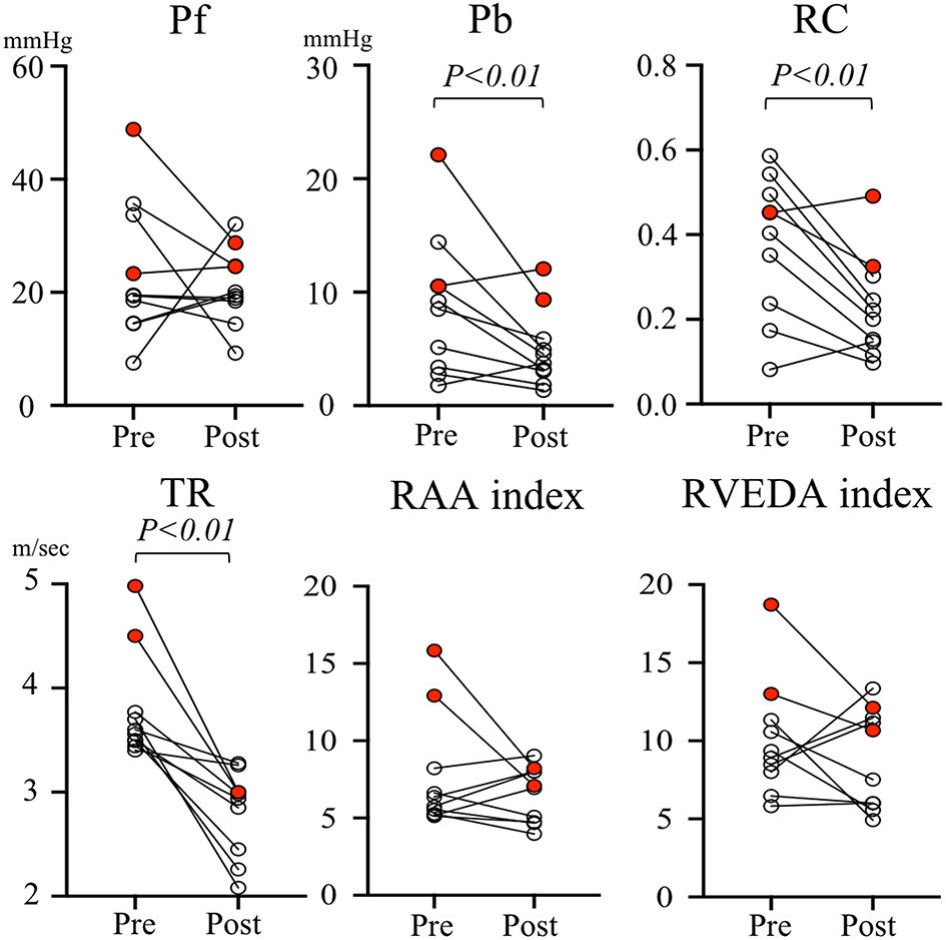
Individual change in pulmonary artery wave reflection parameters and tricuspid valve regurgitation velocity and right atrial area index, right ventricular end-diastolic area index for each dog before and after mitral valvuloplasty. The red dots represent the two dogs in which PH remains after MVP surgery. Pb, backward pressure; Pf, forward pressure; RAA index, right atrial area index; RC, reflection coefficient which is calculated as the ratio of peak Pb to peak Pf; RVEDA index, right ventricular end-diastolic area index; TR, tricuspid valve regurgitation.

Figure 3 represents the individual change in pulmonary artery wave reflection parameters and TR velocity, RAA index, RVEDA index for each dog before and after MVP. The red dots represent the two dogs in which PH remained after MVP surgery. When looking at the individual change in pulmonary artery wave reflection parameters for each dog, Pb and RC were significantly decreased after MVP in almost all dogs. However, two dogs in which PH persisted after MVP (red dots) had higher Pb (12.1 and 9.34 mmHg) and RC (0.49 and 0.32) compared with the other dogs (black dots). On the other hand, TR and RAA index, RVEDA index of these two dogs were not so high compared with the other dogs.

### Correlation Between the Pulmonary Artery Wave Reflection Parameter, Echocardiographic Parameters, and RAP

**Figure 4:**
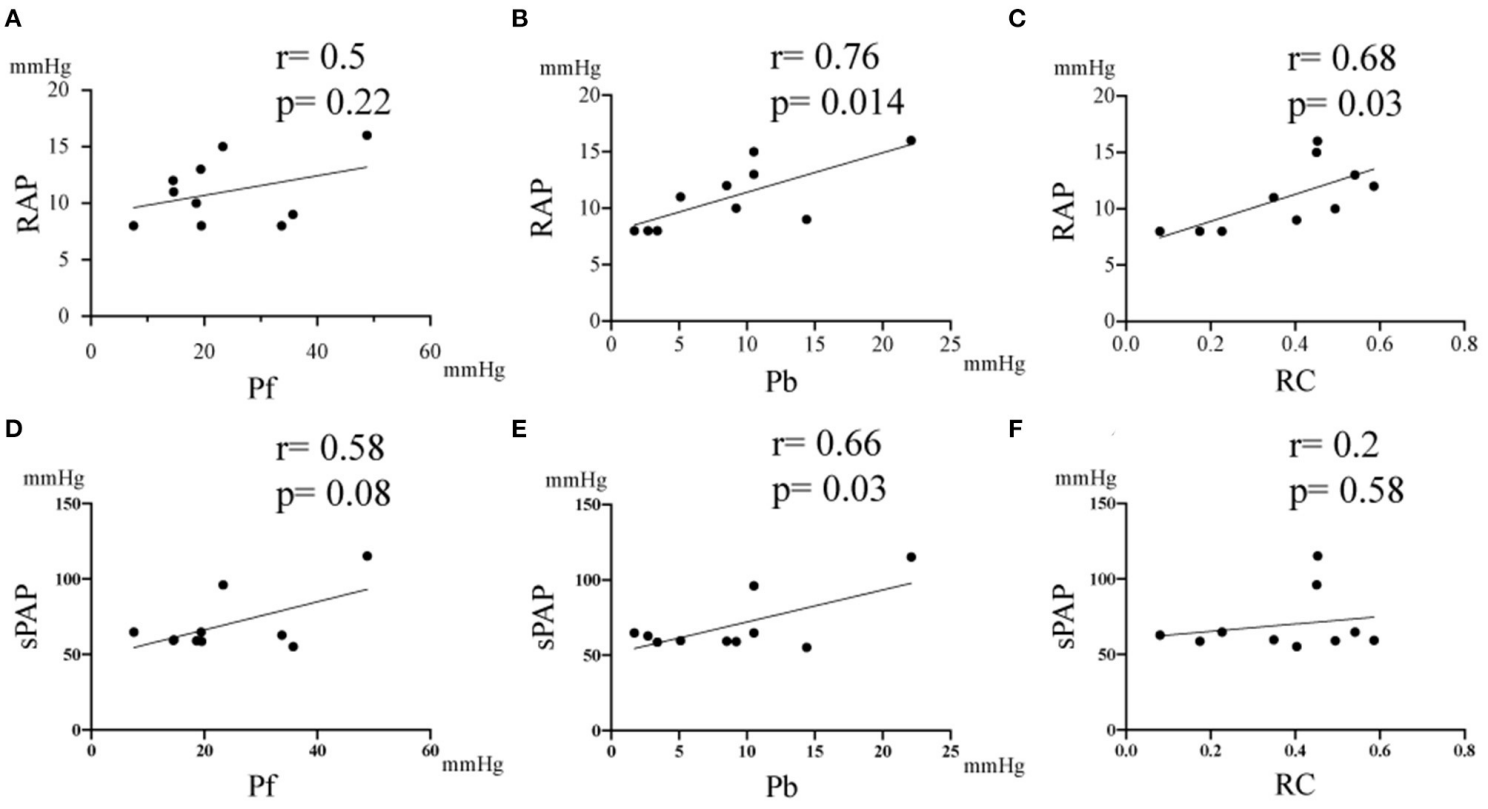
Correlation between the pulmonary artery wave reflection parameter and right atrium pressure (RAP), and estimated systolic pulmonary artery pressure (sPAP). **(A)** Correlation between RAP vs Pf. **(B)** Correlation between RAP vs Pb. **(C)** Correlation between RAP vs RC. **(D)** Correlation between sPAP vs Pf. **(E)** Correlation between Estimated sPAP vs Pb. **(F)** Correlation between Estimated sPAP vs RC. RC, reflection coefficient which is calculated as the ratio of peak Pb to peak Pf; Pb, backward pressure; Pf, forward pressure; r, the correlation coefficient.

Table 3 summarizes the correlation results between the pulmonary artery wave reflection parameters and hemodynamic parameters, and echocardiographic parameters before MVP. There was no correlation between Pf, RC and RAP, Estimated sPAP (Table 3 and Figures 4A,D,F, Pf vs. RAP, *r* = 0.5, *p* = 0.22; Pf vs. Estimated sPAP, *r* = 0.58, *p* = 0.08; RC vs. Estimated sPAP, *r* = 0.2, *p* = 0.58). On the other hands, there was a statistically significant positive correlation between 676 Pb, RC, and RAP, Estimated sPAP (Table 3 and Figures 4B,C,E, Pb vs. RAP, *r* = 0.76, *p* = 0.014; Pb vs. Estimated sPAP, *r* = 0.76, *p* = 0.014; RC vs. RAP, *r* = 0.68, *p* = 0.03). RAP and Estimated sPAP measured during surgery were 11 ± 2.8 mmHg and 69.5 ± 18.73 mmHg. No significant correlation was identified between pulmonary artery wave reflection parameters and other echocardiographic parameters (Table 3).

**Table 3:** Correlation analysis between pulmonary artery wave reflection parameters and hemodynamic and echocardiographic parameters.

	**Pb**		**RC**	
	***R***	***P*** **-value**	***R***	***P*** **-value**
RAP	0.76^*^	0.014	0.68^*^	0.03
Estimated sPAP	0.66^*^	0.035	0.32	0.12
RVOT velocity	0.017	0.96	−0.2	0.59
E velocity	0.1	0.77	−0.03	0.94
LVIDDN	−0.42	0.22	0.15	0.67
E:e′ sep	0.41	0.23	0.2	0.59
E:e′ lat	−0.05	0.88	−0.24	0.5
TR velocity	0.6	0.07	0.1	0.78
RAA index	0.61	0.06	0.19	0.6
RVEDA index	0.33	0.35	0.18	0.6

*E velocity, early diastolic mitral inflow velocity; e′, early diastolic wave signal as measured by Tissue Doppler imaging; lat, mitral annulus at the left ventricular lateral wall; LVIDDN, normalized left ventricular internal dimension in diastole; Pb, backward pressure; RAA index, right atrial area index; RC, reflection coefficient; RVEDA index, right ventricular end-diastolic area index; RVOT, right ventricular outflow tract; RAP, right atrial pressure; Estimated sPAP, estimated systolic pulmonary arterial pressure; sep, mitral annulus at the septal wall; TR velocity, tricuspid regurgitation flow velocity.*

* *p < 0.05*.

### The Effect of Pulmonary Artery Wave Reflection Parameters on Hemodynamic and Echocardiographic Parameters

Result of linear regression analysis of the parameters of pulmonary artery wave reflection, hemodynamic parameters are shown in Table 4. There was a significant effect of Pb on RAP, and Estimated sPAP (Pb vs. RAP, *R*^2^ = 0.54, *p* = 0.015; Pb vs. Estimated sPAP, *R*^2^ = 0.44, *p* = 0.036; RC vs. RAP), respectively. RC demonstrated significant effect on RAP and Estimated sPAP (*R*^2^ = 0.46, *p* = 0.03; RC vs. Estimated sPAP, *R*^2^ = 0.042, *p* = 0.57). Pb and RC did not significantly affect other parameters.

**Table 4:** The effect of pulmonary artery wave reflection parameters on hemodynamic and echocardiographic parameters.

	**Pb**		**RC**	
	***R*** **^2^**	***P*** **-value**	***R*** **^2^**	***P*** **-value**
RAP	0.54^*^	0.015	0.46^*^	0.03
Estimated sPAP	0.44^*^	0.036	0.042	0.57
RVOT velocity	0.0003	0.96	0.04	0.59
E velocity	0.011	0.77	0.0007	0.94
LVIDDN	0.18	0.23	0.023	0.67
E:e′ sep	0.17	0.24	0.039	0.59
E:e′ lat	0.003	0.88	0.058	0.5
TR velocity	0.36	0.065	0.01	0.78
RAA index	0.374	0.06	0.03	0.06
RVEDA index	0.10	0.36	0.03	0.61

*E velocity, early diastolic mitral inflow velocity; e′, early diastolic wave signal as measured by Tissue Doppler imaging; lat, mitral annulus at the left ventricular lateral wall; LVIDDN, normalized left ventricular internal dimension in diastole; Pb, backward pressure; RAA index, right atrial area index; RC, reflection coefficient; RVEDA index, right ventricular end-diastolic area index; RVOT, right ventricular outflow tract; RAP, right atrial pressure; Estimated sPAP, estimated systolic pulmonary arterial pressure; sep, mitral annulus at the septal wall; TR velocity, tricuspid regurgitation flow velocity.*

* *p < 0.05*.

## Discussion

This study investigated both conventional echocardiographic parameters and pulmonary artery wave reflection parameters before and after MVP in patients with PH due to MMVD. Results indicated pulmonary artery wave reflection was closely related to PH that persisted despite treatment.

In recent years, cardiac surgery techniques in the small animal field have made great strides and MVP has contributed significantly to lowering left atrial pressure by improving mitral regurgitation caused by MMVD (35–37). Reduction of left atrial pressure by MVP is one of the treatments for secondary PH caused by MMVD, as a persistent increase in left atrial pressure induces PH (38, 39). MVP could be an effective treatment for MMVD with PH due to decreasing the following parameters after surgery: LVIDDN; LA/Ao; E velocity; E′ sep; E′ lat; TR velocity and wave reflection. In two cases, higher wave reflection was observed both before and after MVP, with the RC diminished only slightly following MVP in one dog, however, RC increased slightly in the other one. This finding suggests that despite improving the left atrial pressure caused by left heart failure after MVP, the remodeling of pulmonary artery vascular caused reactive PH in some patient. This may be indicative of residual pulmonary arterial disease, which may continue to adversely affect interactions between the right ventricle and the vasculature.

Pulmonary artery wave reflection occurs when the forward flow out of the right ventricle is reflected by the pulmonary arterial tree, generating a backward wave. Pulmonary artery wave reflection can be detected by measuring pulmonary artery flow and pressure simultaneously using wave intensity analysis. In particular, wave reflection is enhanced when the energy transmission property changes between the proximal and distal vasculature due to vascular remodeling, arteriosclerosis, or thrombus (20, 24, 40). Pulmonary artery wave reflection can potentially provide novel information regarding pulmonary hemodynamics to supplement traditional methods used to evaluate PH, such as PAP and PVR, which are the most common hemodynamic measurements used to evaluate its progression (20, 41, 42). Wave reflection is not typically applied in the small animal clinical setting as this requires catheterization. In this study, we could measure wave reflection non-invasively in MMVD cases with PH, as in recent years, wave reflection has been calculated non-invasively through Doppler echocardiography (26).

In eight out of 10 cases, the post-operative Pb and RC decreased significantly compared with pre-operative measurements. This was attributed to the decrease in pulmonary arterial pressure and the pulmonary vascular resistance, through improving the pulmonary vein pressure by MVP. However, the Pb and RC of two cases in which pulmonary hypertension remained after MVP had higher Pb and RC than the other eight cases, suggesting that the wave reflection did not improve. It could be that the two cases may have already progressed to reactive post-capillary-PH, due to remodeling of the pulmonary artery. Therefore, we suggest that pulmonary hypertension remained and the wave reflection was a high value, despite the pulmonary vein pressure decreasing after MVP. Two dogs with persistent PH after MVP presented with flattened interventricular septum despite mild TR flow velocity (TR velocity 3.0 m/s in both PH patient). Although the measurement of estimated pulmonary artery systolic pressure using TR is often used as a screening tool for the diagnosis and severity of PH, the accuracy of this method has been questioned because it is affected by right ventricular dysfunction and technical errors (43, 44). Thus, it may not be possible to detect the presence of persistent PH simply by measuring the TR flow velocity. This study also indicated that measurement of the RAA index and RVEDA index couldn't detect the presence of persistent PH. Since the RAA index and RVEDA index are affected by changes in left ventricular volume, size of right heart is not always associated with severity of PH. On the other hand, analysis of pulmonary artery wave reflection could help detection of persistent PH.

Su et al. previously demonstrated that in patients with chronic thromboembolic pulmonary hypertension, both a large wave reflection and symptoms related to PH remained and were not improved despite pulmonary endarterectomy being performed (45). Despite successful surgery, the sustained and potentially irreversible impact of vascular remodeling could contribute to persistent vascular admittance mismatching. Persistent vascular admittance mismatching leads to higher wave reflection. Therefore, we concluded that it is useful to measure pulmonary artery wave reflection in PH due to MMVD before and after MVP, since pulmonary artery wave reflection, as determined by wave intensity analysis, may provide additional information about assessing pulmonary hemodynamics. Interestingly, Pb correlates with right atrial pressure and systolic pulmonary artery pressure, suggesting that wave reflection may be an indicator of congestion of the right heart and right ventricular afterload. On the other hand, RC represents the ratio of wave reflection pressure to forward wave pressure. It has been reported that RC is an index of right ventricular function and right ventricular afterload, indicating that it may not simply correlate with pulmonary arterial pressure (20, 26). Thus, caution is required in interpreting this result.

In conclusion, both before and after MVP in PH patients with left heart failure, high wave reflection was observed. The magnitude of wave reflection diminished in almost all patients after MVP. From the results, we conclude that MVP is one of the treatment methods for secondary PH due to MMVD. Through this method post-operative echocardiographic and wave reflection parameters can improve and the right afterload can be reduced. In some patients, however, despite improving the left atrial pressure due to left heart failure after MVP, some degree of vascular admittance mismatch persisted. This may be indicative of residual pulmonary arterial disease, which may continue to adversely affect interactions between the right ventricle and the vasculature.

## Limitations

In this study, the number of PH due to MMVD cases was small; therefore, additional studies should be performed. PH may not have been accurately evaluated, given pulmonary vascular resistance and pulmonary artery wedge pressure—the gold standards for evaluating the hemodynamic assessment of PH—were not measured using a catheter. The value of the pulmonary artery wave reflection may differ from that measured by a catheter since the measurement method is by non-invasive Doppler echocardiography. In this non-invasive method using echocardiography, since pressure and flow velocity profile are gained at different timing, pulmonary arterial wave reflection may be affected by situation of examined dogs (exciting level) and respiration timing. In clinical cases, measuring the pulmonary artery wave reflection in cases that do not exhibit TR can be difficult, as can the evaluation of wave reflection in cases that present with severe arrhythmia. Right ventricular function (TAPSE, tricuspid annular plane systolic excursion; RV-STE, right ventricular speckle tracking, etc.) wasn't measured in this study. However, we think the indicator of right ventricular function cannot be evaluated correctly after surgery. After MVP, heart function decreases temporarily due to invasive open-heart surgery. In addition, cardiac contractility decreases ostensibly due to reducing the volume loading by improved mitral regurgitation. Therefore, relationship between pulmonary arterial wave reflection and right ventricular function are unclear. In the future, it is necessary to investigate the relationship between wave reflection and the right ventricular function.

The scale of this study was small and that limits being able to affirm that the technique has been validated completely. More animal research and subsequent research in humans are necessary to clinically apply this novel indicator.

## Data Availability Statement

The raw data supporting the conclusions of this article will be made available by the authors, without undue reservation.

## Ethics Statement

The animal study was reviewed and approved by Shiraishi Animal Hospital Animal Care and Use Committee (protocol number R2-062). Written informed consent was obtained from the owners for the participation of their animals in this study.

## Author Contributions

TY and KM designed the study and prepared original draft. KS acquired and analyzed the data. LH interpreted the results and critically reviewed the results. RT and TU edited the manuscript and approved the final version of the manuscript. All authors contributed to the article and approved the submitted version.

## Conflict of Interest

The authors declare that the research was conducted in the absence of any commercial or financial relationships that could be construed as a potential conflict of interest.

## Publisher's Note

All claims expressed in this article are solely those of the authors and do not necessarily represent those of their affiliated organizations, or those of the publisher, the editors and the reviewers. Any product that may be evaluated in this article, or claim that may be made by its manufacturer, is not guaranteed or endorsed by the publisher.

## Abbreviations

AcT: acceleration time
ACVIM: American college of veterinary internal medicine
AO: aortic
BSA: body surface area
CTEPH: chronic thromboembolic pulmonary hypertension
ET: ejection time
LVIDDN: normalized left ventricular internal diameter at end-diastole
MMVD: myxomatous mitral valve disease
MPA: main pulmonary artery
MVP: mitral valvuloplasty
LVIDd: left ventricular internal dimension in diastole
P: pressure
PAH: pulmonary artery hypertension
PAP: pulmonary artery pressure
Pb: backward pressure
Pf: forward pressure
PH: pulmonary hypertension
PVR: pulmonary vascular resistance
RAA index: right atrial area index
RAP: right atrium pressure
RC: reflection coefficient
ROC: receiver-operating characteristic
RVEDA index: right ventricular end-diastolic area index
RVOT: right ventricular outflow tract
SD: standard deviation
TR: tricuspid valve regurgitation
U: velocity
WRI: wave reflection indices.

## References

[1] Buchanan J. Chronic valvular disease (endocardiosis) in dogs. Adv Vet Sci Comp Med. (1977) 21:75–106. 146409

[2] Serres F Chetboul V Tissier R Sampedrano CC Gouni V Nicolle AP. Chordae tendineae rupture in dogs with degenerative mitral valve disease: prevalence, survival, and prognostic factors (114 cases, 2001–2006). J Vet Int Med. (2007) 21:258–64. 10.1892/0891-6640(2007)21[258:CTRIDW]2.0.CO;2 17427386

[3] Boswood A Gordon S Häggström J Wess G Stepien R Oyama M. Longitudinal analysis of quality of life, clinical, radiographic, echocardiographic, and laboratory variables in dogs with preclinical myxomatous mitral valve disease receiving pimobendan or placebo: the EPIC study. J Vet Int Med. (2018) 32:72–85. 10.1111/jvim.14885 29214723 PMC5787203

[4] Borgarelli M Abbott J Braz-Ruivo L Chiavegato D Crosara S Lamb K. Prevalence and prognostic importance of pulmonary hypertension in dogs with myxomatous mitral valve disease. J Vet Int Med. (2015) 29:569–74. 10.1111/jvim.12564 25818210 PMC4895522

[5] Tidholm A Höglund K Häggström J Ljungvall I. Diagnostic value of selected echocardiographic variables to identify pulmonary hypertension in dogs with myxomatous mitral valve disease. J Vet Int Med. (2015) 29:1510–7. 10.1111/jvim.13609 26365438 PMC4895671

[6] Reinero C Visser LC Kellihan HB Masseau I Rozanski E Clercx C. ACVIM consensus statement guidelines for the diagnosis, classification, treatment, and monitoring of pulmonary hypertension in dogs. J Vet Int Med. (2020) 34:549–73. 10.1111/jvim.15725 32065428 PMC7097566

[7] Galiè N Humbert M Vachiery J-L Gibbs S Lang I Torbicki A. 2015 ESC/ERS guidelines for the diagnosis and treatment of pulmonary hypertension: the joint task force for the diagnosis and treatment of pulmonary hypertension of the European Society of Cardiology (ESC) and the European Respiratory Society (ERS): endorsed by: Association for European Paediatric and Congenital Cardiology (AEPC), International Society for Heart and Lung Transplantation (ISHLT). Eur Heart J. (2016) 37:67–119. 10.1093/eurheartj/ehv317 26320113

[8] Pfeuffer-Jovic E Weiner S Wilkens H Schmitt D Frantz S Held M. Impact of the new definition of pulmonary hypertension according to world symposium of pulmonary hypertension 2018 on diagnosis of post-capillary pulmonary hypertension. Int J Cardiol. (2021) 335:105–10. 10.1016/j.ijcard.2021.04.006 33823213

[9] Fukumoto S Hanazono K Miyasho T Endo Y Kadosawa T Iwano H. Serum big endothelin-1 as a clinical marker for cardiopulmonary and neoplastic diseases in dogs. Life Sci. (2014) 118:329–32. 10.1016/j.lfs.2014.01.002 24447631

[10] Tatebe S Fukumoto Y Sugimura K Miyamichi-Yamamoto S Aoki T Miura Y. Clinical significance of reactive post-capillary pulmonary hypertension in patients with left heart disease. Circ J. (2012) 76:1235–44. 10.1253/circj.CJ-11-1288 22313804

[11] Gerges M Gerges C Pistritto A-M Lang MB Trip P Jakowitsch J. Pulmonary hypertension in heart failure. Epidemiology, right ventricular function, and survival. Am J Respir Crit Care Med. (2015) 192:1234–46. 10.1164/rccm.201503-0529OC 26181215

[12] Vevecka AH Uerban M Jurcut R Ginghină C. Reactive pulmonary hypertension in left heart disease “post-capillary PH with a pre-capillary component”. Romanian J Cardiol. (2015) 25:147–52.

[13] Ibe T Wada H Sakakura K Ugata Y Maki H Yamamoto K. Combined pre-and post-capillary pulmonary hypertension: the clinical implications for patients with heart failure. PLoS ONE. (2021) 16:e0247987. 10.1371/journal.pone.0247987 33651852 PMC7924774

[14] Enriquez-Sarano M Akins CW Vahanian A. Mitral regurgitation. Lancet. (2009) 373:1382–94. 10.1016/S0140-6736(09)60692-9 19356795

[15] Walls MC Cimino N Bolling SF Bach DS. Persistent pulmonary hypertension after mitral valve surgery: does surgical procedure affect outcome? J Heart Valve Dis. (2008) 17:1–9. 18365562

[16] Paşaoglu I Demircin M Dogan R Ozmen F Ersoy U Böke E. Mitral valve surgery in the presence of pulmonary hypertension. Jpn Heart J. (1992) 33:179–84. 10.1536/ihj.33.179 1593747

[17] Murashita T Okada Y Kanemitsu H Fukunaga N Konishi Y Nakamura K. The impact of preoperative and postoperative pulmonary hypertension on long-term surgical outcome after mitral valve repair for degenerative mitral regurgitation. Ann Thorac Cardiovasc Surg. (2015) 21:53–8. 10.5761/atcs.oa.13-00364 24747547 PMC4989987

[18] Jegaden O Rossi R Delahaye F Montagna P Delaye J Delahaye JP. Mitral valve replacement in severe pulmonary hypertension. Long-term results. Archives des Maladies du Coeur et des Vaisseaux. (1991) 84:1297–301. 1958113

[19] Uechi M. Mitral valve repair in dogs. J Vet Cardiol. (2012) 14:185–92. 10.1016/j.jvc.2012.01.004 22366571

[20] Su J Manisty C Parker KH Simonsen U Nielsen-Kudsk JE Mellemkjaer S. Wave intensity analysis provides novel insights into pulmonary arterial hypertension and chronic thromboembolic pulmonary hypertension. J Am Heart Assoc. (2017) 6:e006679. 10.1161/JAHA.117.006679 29089339 PMC5721764

[21] Castelain V Hervé P Lecarpentier Y Duroux P Simonneau G Chemla D. Pulmonary artery pulse pressure and wave reflection in chronic pulmonary thromboembolism and primary pulmonary hypertension. J Am Coll Cardiol. (2001) 37:1085–92. 10.1016/S0735-1097(00)01212-2 11263613

[22] Jones CJ Sugawara M Kondoh Y Uchida K Parker KH. Compression and expansion wavefront travel in canine ascending aortic flow: wave intensity analysis. Heart Vessels. (2002) 16:91–8. 10.1007/s003800200002 12027238

[23] Parker KH Jones C. Forward and backward running waves in the arteries: analysis using the method of characteristics. J Biomech Eng. (1990) 112(3):322–6. 10.1115/1.2891191 2214715

[24] Hollander EH Wang J-J Dobson GM Parker KH Tyberg JV. Negative wave reflections in pulmonary arteries. Am J Physiol Heart Circ Physiol. (2001) 281:895–H902. 10.1152/ajpheart.2001.281.2.H895 11454596

[25] Fukumitsu M Kawada T Shimizu S Turner MJ Uemura K Sugimachi M. Wave reflection correlates with pulmonary vascular wall thickening in rats with pulmonary arterial hypertension. Int J Cardiol. (2017) 249:396–401. 10.1016/j.ijcard.2017.09.024 28939268

[26] Yoshida T Matsuura K Seijirow G Uemura A Yilmaz Z Tanaka R. Non-invasive assessment of pulmonary artery wave reflection in dogs with suspected pulmonary hypertension. Front Vet Sci. (2021) 8:659194. 10.3389/fvets.2021.659194 34307519 PMC8298900

[27] Westerhof N Sipkema P Bos GVD Elzinga G. Forward and backward waves in the arterial system. Cardiovasc Res. (1972) 6:648–56. 10.1093/cvr/6.6.648 4656472

[28] Li JK-J. Time domain resolution of forward and reflected waves in the aorta. IEEE Transac Biomed Eng. (1986) 33:783–5. 10.1109/TBME.1986.325903 3744394

[29] Khir A O'brien A Gibbs J Parker K. Determination of wave speed and wave separation in the arteries. J Biomech. (2001) 34:1145–55. 10.1016/S0021-9290(01)00076-8 11506785

[30] Cornell CC Kittleson MD Torre PD Häggström J Lombard CW Pedersen HD. Allometric scaling of M-mode cardiac measurements in normal adult dogs. J Vet Int Med. (2004) 18:311–21. 10.1111/j.1939-1676.2004.tb02551.x 15188817

[31] Boon JA. Veterinary Echocardiography. Fort Collins: John Wiley & Sons (2011).

[32] Pyle RL Abbott J MacLean H. Pulmonary hypertension and cardiovascular sequelae in 54 dogs. Intern J Appl Res Vet Med. (2004) 2:99–109.

[33] Visser LC Im M Johnson LR Stern JA. Diagnostic value of right pulmonary artery distensibility index in dogs with pulmonary hypertension: comparison with Doppler echocardiographic estimates of pulmonary arterial pressure. J Vet Int Med. (2016) 30:543–52. 10.1111/jvim.13911 26893108 PMC4913611

[34] Serres F Chetboul V Gouni V Tissier R Sampedrano CC Pouchelon JL. Diagnostic value of echo-Doppler and tissue Doppler imaging in dogs with pulmonary arterial hypertension. J Vet Int Med. (2007) 21:1280–9. 10.1111/j.1939-1676.2007.tb01950.x 18196738

[35] Kanemoto I Taguchi D Yokoyama S Mizuno M Suzuki H Kanamoto T. Open heart surgery with deep hypothermia and cardiopulmonary bypass in small and toy dogs. Vet Surg. (2010) 39:674–9. 10.1111/j.1532-950X.2010.00687.x 20459489

[36] Kanemoto I Mihara K Sato K. Open-heart techniques and mitral valve plasty for mitral regurgitation in toy-and small-breed dogs: a review. Open Vet J. (2021) 11:14–26. 10.4314/ovj.v11i1.4 33898279 PMC8057224

[37] Uechi M Mizukoshi T Mizuno T Mizuno M Harada K Ebisawa T. Mitral valve repair under cardiopulmonary bypass in small-breed dogs: 48 cases (2006–2009). J Am Vet Med Assoc. (2012) 240:1194–201. 10.2460/javma.240.10.1194 22559109

[38] Ward C Hancock B. Extreme pulmonary hypertension caused by mitral valve disease. Natural history and results of surgery. Heart. (1975) 37:74–8. 10.1136/hrt.37.1.74 1111561 PMC484156

[39] Zener JC Hancock EW Shumway NE Harrison DC. Regression of extreme pulmonary hypertension after mitral valve surgery. Am J Cardiol. (1972) 30:820–6. 10.1016/0002-9149(72)90005-7 4634279

[40] Bouwmeester JC Belenkie I Shrive NG Tyberg JV. Wave reflections in the pulmonary arteries analysed with the reservoir–wave model. J Physiol. (2014) 592:3053–62. 10.1113/jphysiol.2014.273094 24756638 PMC4214659

[41] Quail MA Knight DS Steeden JA Taelman L Moledina S Taylor AM. Noninvasive pulmonary artery wave intensity analysis in pulmonary hypertension. Am J Physiol Heart Circ Physiol. (2015) 308:1603–H11. 10.1152/ajpheart.00480.2014 25659483 PMC4469876

[42] Su J Hughes AD Simonsen U Howard LS. Nitric oxide attenuates arterial pulse wave reflection in a vasodilator responding pulmonary arterial hypertension patient. Circ Cardiovasc Interv. (2018) 11:e006242. 10.1161/CIRCINTERVENTIONS.117.006242 29870387

[43] Fisher MR Forfia PR Chamera E Housten-Harris T Champion HC Girgis RE. Accuracy of Doppler echocardiography in the hemodynamic assessment of pulmonary hypertension. Am J Respir Crit Care Med. (2009) 179:615–21. 10.1164/rccm.200811-1691OC 19164700 PMC2720125

[44] Mutlak D Aronson D Lessick J Reisner SA Dabbah S. Agmon Functional tricuspid regurgitation in patients with pulmonary hypertension: is pulmonary artery pressure the only determinant of regurgitation severity? Chest. (2009) 135:115–21. 10.1378/chest.08-0277 18719061

[45] Su J Hughes AD Simonsen U Nielsen-Kudsk JE Parker KH Howard LS. Impact of pulmonary endarterectomy on pulmonary arterial wave propagation and reservoir function. Am J Physiol Heart Circ Physiol. (2019) 317:505–H16. 10.1152/ajpheart.00181.2019 31225986 PMC6703995

